# *sut-2* loss of function mutants protect against tau-driven shortened lifespan and hyperactive pharyngeal pumping in a *C. elegans* model of tau toxicity

**DOI:** 10.17912/micropub.biology.000844

**Published:** 2023-08-03

**Authors:** Heather N Currey, Brian C. Kraemer, Nicole F Liachko

**Affiliations:** 1 Geriatric Research Education and Clinical Center, VA Puget Sound Health Care System, Seattle, Washington, United States; 2 Department of Medicine, Division of Gerontology and Geriatric Medicine, University of Washington, Seattle, Washington, United States; 3 Department of Psychiatry and Behavioral Sciences, University of Washington, Seattle, Washington, United States; 4 Department of Laboratory Medicine and Pathology, University of Washington, Seattle, Washington, United States

## Abstract

Expression of human tau in *C. elegans* neurons causes progressive, age-associated loss of motor coordination, selective neurodegeneration, and shortened lifespan. Loss of function (LOF) mutations in the conserved gene *sut-2* protects against progressive motor uncoordination and neurodegeneration in models of tauopathy. To determine whether *sut-2* LOF also protects against shortened lifespan of tau transgenic *C. elegans* , we conducted lifespan assays comparing four different alleles of *sut-2 .* We found that *sut-2* LOF robustly suppresses the shortened lifespan of tau transgenic animals. We also demonstrate that tau transgenic *C. elegans* exhibit hyperactive pharyngeal pumping, which is restored by *sut-2* LOF.

**Figure 1. sut-2 loss of function mutants suppress tau Tg lifespan and pharyngeal pumping phenotypes:**
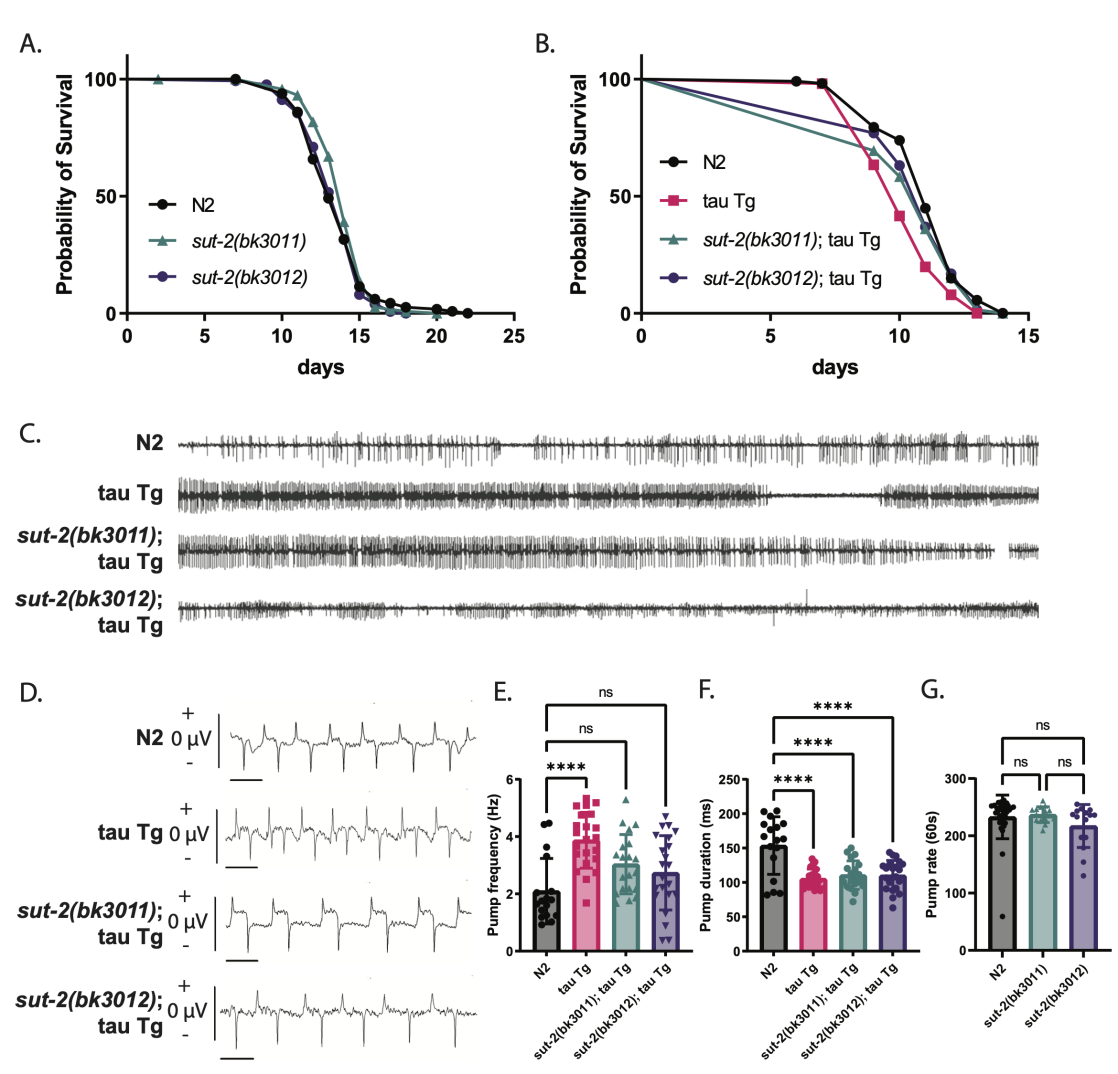
A) *sut-2 (bk3011)* and *sut-2 (bk3012)* have a similar lifespan to N2 (wild-type control animals). B) *sut-2 (bk3011)* and *sut-2 (bk3012)* rescue the shortened lifespan of tau Tg animals. See Table 1 for lifespan numbers (N), mean, and p-values. C) Time-compressed recordings of *C. elegans* pumping activity over two minutes. Each line spike above and below the center describes the action potential of a single pump. tau Tg animals pump more often than N2 , represented by a denser trace. *sut-2 (bk3011);* tau Tg and *sut-2 (bk3012);* tau Tg pumping is similar to that of N2 . D) Expanded view of pharyngeal action potentials showing both positive (excitatory) and negative (relaxation) spikes, which delineate the beginning and end of a single action potential. The scale bar represents 200 ms of recording. E) tau Tg animals have significantly increased pump frequency compared to N2 , p<0.0001. *sut-2 (bk3011);* tau Tg, and *sut-2 (bk3012);* tau Tg pump frequencies are not significantly different from N2 . Average pump frequency over a 2 minute recording, N=17 for N2 , N=22 for tau Tg, N=20 for *sut-2 (bk3011);* tau Tg, and N=22 for *sut-2 (bk3012);* tau Tg. Significance was evaluated using one-way analysis of variance with Tukey’s multiple comparison test. F) tau Tg, *sut-2 (bk3011);* tau Tg, and *sut-2 (bk3012);* tau Tg animals have significantly decreased pump durations compared to N2 , p<0.0001. Average pump duration over a 2 minute recording, N=17 for N2 , N=22 for tau Tg, N=20 for *sut-2 (bk3011);* tau Tg, and N=22 for *sut-2 (bk3012);* tau Tg . Significance was evaluated using one-way analysis of variance with Tukey’s multiple comparison test. G) *sut-2 (bk3011)* and *sut-2 (bk3012)* have similar pumping frequencies to N2 . Average pharyngeal pumps per minute was assessed with manual counting, N=30 for N2 , N=15 for *sut-2 (bk3011)* , and N=15 for *sut-2 (bk3012)* .

## Description

Pathological inclusions of the protein tau in neurons and glia characterize several human neurodegenerative diseases including frontotemporal lobar degeneration (FTLD-tau), progressive supranuclear palsy (PSP), chronic traumatic encephalopathy (CTE), and Alzheimer’s disease (Limorenko and Lashuel 2022) . To model disease-associated tau, transgenic *C. elegans* have been generated that express human 1N4R tau with the V337M FTLD-tau causative mutation (tau(V337M)) in all neurons (tau Tg). These animals exhibit uncoordinated movement, age-dependent neurodegeneration, and shortened lifespan (Kraemer, Zhang et al. 2003) . Forward genetic screening identified mutations in the gene *sut-2* that suppress these phenotypes (Guthrie, Schellenberg et al. 2009, Guthrie, Greenup et al. 2011) . Subsequent CRISPR generated null mutations that deleted the entire *sut-2* coding sequence, *sut-2 (bk3011)* and *sut-2 (bk3012),* were found to similarly suppress tau movement dysfunction and neurodegeneration (Kow, Strovas et al. 2021, Latimer, Stair et al. 2022) .

The *sut-2* LOF mutants ( *sut-2 (bk3011)* and *sut-2 (bk3012)* ) have lifespan similar to N2 ( **Fig 1A** and **Table 1** ). To test whether *sut-2 (bk3011)* and *sut-2 (bk3012)* mutations are able to rescue the shortened lifespan of tau transgenic worms similarly to previously characterized alleles of *sut-2* , *sut-2 ( bk87 )* and *sut-2 ( bk741 )* , we measured the lifespans of tau transgenic animals crossed to these four *sut-2* mutant alleles. In fact, we find that *sut-2 (bk3011)* and *sut-2 (bk3012)* robustly suppress the shortened lifespan of tau Tg animals similar to *sut-2 ( bk87 )* and *sut-2 ( bk741 )* ( **Fig 1B** and **Table 1** ).

To test whether tau Tg expressing *C. elegans* exhibit altered pharyngeal pumping, we assayed pharyngeal muscles and neurons electrophysiology from individual animals using a microfluidic chip-based recording device. This device detects, records, and evaluates pharyngeal muscle and neuron action potentials that accompany each pump cycle. We found tau Tg animals had significantly increased frequency and reduced duration of pharyngeal pumping ( **Fig 1C-F** ). We then tested whether *sut-2 (bk3011)* and *sut-2 (bk3012)* can modify tau Tg *C. elegans* pumping defects. We found that *sut-2 (bk3011)* and *sut-2 (bk3012)* partially suppress the increased frequency but do not suppress the decreased duration of pumping in tau Tg animals ( **Fig 1C-F** ). *sut-2 (bk3011)* and *sut-2 (bk3012)* do not have altered pumping rates relative to N2 ( **Fig 1G** ).

Taken together, these data show that complete elimination of *sut-2* via a whole gene deletion ameliorates the toxic consequence of tauopathy in tau transgenic *C. elegans* . *sut-2* and its mammalian homolog MSUT2 may be compelling targets to treat tauopathies including Alzheimer’s disease.

**Table 1**

**Table:** 

**Experiment 1**	**N2**	**Tau tg**	***sut-2 ( bk87 )* ; Tau Tg **	***sut-2 ( bk741 )* ; Tau Tg **
**N**	154	124	119	112
**Mean**	12.92	11.48	12.48	14.18
** p-value ( N2 ) **	-	<0.0001	N.S.	<0.0001
**p-value (Tau Tg)**	-	-	<0.0001	<0.0001
**Experiment 2**	**N2**	**Tau tg**	***sut-2 (bk3011)* ; Tau Tg **	***sut-2 (bk3012)* ; Tau Tg **
**N**	107	101	72	65
**Mean**	11.14	10.29	10.81	10.95
** p-value ( N2 ) **	-	<0.0001	N.S.	N.S.
**p-value (Tau Tg)**	-	-	0.0163	0.0045
**Experiment 3**	**N2**	**Tau tg**	***sut-2 (bk3011)* ; Tau Tg **	***sut-2 (bk3012)* ; Tau Tg **
**N**	114	118	121	119
**Mean**	13.56	13.03	13.79	14.16
** p-value ( N2 ) **	-	0.017	N.S.	<0.0001
**p-value (Tau Tg)**	-	-	0.0001	<0.0001
**Experiment 4**	**N2**	***sut-2 (bk3011) ***	***sut-2 (bk3012) ***	
**N**	114	115	124	
**Mean**	13.56	13.97	13.4	
** p-value ( N2 ) **	-	N.S.	N.S.	

**Table legend**

N = number tested, Mean = average population lifespan in days, p-value ( N2 ) = significance relative to N2 (wild-type control), pvalue (tau Tg) = significance relative to tau Tg animals. N.S. = not significant. Significance was evaluated using survival curve comparisons with Mantel-cox log-rank analysis.

## Methods

*C. elegans* lifespan assays

Lifespan assays were modified from those described in (Liachko, Davidowitz et al. 2009) . In brief, worms were grown at 25 ^o^ C following a short (4-6 hour) egglay to L4 stage on NGM plates seeded with *E. coli* OP50 , and then transferred onto seeded NGM plates with added 5-fluoro-2’-deoxyuridine (FUDR, 0.05 mg/mL) to inhibit growth of progeny. Worms were scored every 1-2 days for movement following tapping of the plate or gentle touching with a platinum wire. Failure to respond to touch was scored as dead. Statistical analysis was performed using GraphPad Prism software.

*C. elegans* pumping assays

*C. elegans* pumping electrophysiology was evaluated using a ScreenChip System (NemaMetrix/ InVivo Biosystems) as described in (Latimer, Stair et al. 2022) .In brief, day 1 adult *C. elegans* were pre-incubated in M9 buffer containing 10 mM 5HT (Sigma) for 20 minutes, to stimulate pharyngeal pumping (Song and Avery 2012) . *C. elegans* were then individually loaded into the microfluidic recording device and pharyngeal muscle and neuron action potentials were recorded for two minutes using NemAquire software (NemaMetrix/ InVivo Biosystems).Action potential statistics and readouts, including frequency and duration, were computed using NemAnalysis software (NemaMetrix/ InVivo Biosystems). *C. elegans* pharyngeal pumping rate assay was adapted from (O'Brien 2022). The pump rates were determined by manually counting the number of grinder movements observed in individual animals over 20 seconds. Each animal was recorded 3 times and the average of the 3 recordings was used calculate pumps per minute. Data was managed in Microsoft Excel and statistical analysis was performed using GraphPad Prism Software.

## Reagents

*Strains Used*

N2 Bristol

CK10 *bkIs10* [ *aex-3p* :: *tau(V337M 4R1N);* *myo-2p::GFP* ] III

CK3011 *sut-2 (bk3011)*

CK3012 *sut-2 (bk3012)*

CK1341 *sut-2 (bk3011)* ; *bkIs10* [ *aex-3p* :: *tau(V337M 4R1N);* *myo-2p::GFP* ] III

CK1342 *sut-2 (bk3012)* ; *bkIs10* [ *aex-3p* :: *tau(V337M 4R1N);* *myo-2p::GFP* ] III

CK185 *sut-2 ( bk87 )* ; *bkIs10* [ *aex-3p* :: *tau(V337M 4R1N);* *myo-2p::GFP* ] III

CK187 *sut-2 ( bk741 )* ; *bkIs10* [ *aex-3p* :: *tau(V337M 4R1N);* *myo-2p::GFP* ] III
